# Association between Traffic Air Pollution and Reduced Forced Vital Capacity: A Study Using Personal Monitors for Outdoor Workers

**DOI:** 10.1371/journal.pone.0163225

**Published:** 2016-10-06

**Authors:** Ubiratan Paula Santos, Maria Lúcia Siqueira Bueno Garcia, Alfésio Luís Ferreira Braga, Luiz Alberto Amador Pereira, Chin An Lin, Paulo Afonso de André, Carmen Diva Saldiva de André, Julio da Motta Singer, Paulo Hilário Nascimento Saldiva

**Affiliations:** 1 Pulmonary Division of Heart Institute (InCor) do Hospital das Clínicas da Faculdade de Medicina da Universidade de São Paulo, São Paulo, Brazil; 2 Laboratory of Experimental Therapeutics, Faculdade de Medicina da Universidade de São Paulo, São Paulo, Brazil; 3 Environmental Epidemiology Study Group, Laboratory of Experimental Air Pollution, Faculdade de Medicina da Universidade de São Paulo, São Paulo, Brazil; 4 Environmental Exposure and Risk Assessment Group, Collective Health Post-Graduation Program, Catholic University of Santos, Santos, Brazil; 5 Department of Internal Medicine, Faculdade de Medicina da Universidade de São Paulo, São Paulo, Brazil; 6 Laboratory of Experimental Air Pollution, Department of Pathology, Faculdade de Medicina da Universidade de São Paulo, Brazil; 7 Institute of Mathematics and Statistics, Universidade de São Paulo, São Paulo, Brazil; Northwestern University, UNITED STATES

## Abstract

**Background:**

The effects of outdoor air pollution on lung function in adults are still controversial.

**Objective:**

Evaluate the effects of exposure to different levels of traffic-generated PM_2.5_ on workers’ lung functions in São Paulo, Brazil.

**Methods:**

To cover a wide range of exposures, 101 non-smoking workers from three occupations (taxi drivers, traffic controllers, and forest rangers) were selected for the study. After clinical evaluation, the participants were scheduled to attend four consecutive weekly visits in which they received a 24-hour personal PM_2.5_ sampler and had lung function tests measured on the following day. The association between the spirometric variables and the averaged PM_2.5_ levels was assessed using robust regression models adjusted for age, waist circumference, time at the job, daily work hours, diabetes or hypertension and former smoking habits.

**Results:**

Relative to workers in the lowest exposed group (all measures < 25 μg/m^3^), those with the highest level of exposure (all measures > 39.6 μg/m^3^) showed a reduction of predicted FVC (-12.2%; CI 95%: [-20.0% to -4.4%]), a marginal reduction of predicted FEV_1_ (-9.1%; CI 95%: [-19.1% to 0.9%]) and an increase of predicted FEF_25-75%_/FVC (14.9%; CI 95%: [2.9% to 26.8%]) without changes of FEV_1_/FVC.

**Conclusions:**

Exposure to vehicular traffic air pollution is associated with a small but significant reduction of FVC without a reduction of FEV_1_/FVC.

## Introduction

Ambient air pollution has an important impact on morbidity and mortality. The World Health Organization ascribed 3.7 million deaths to environmental pollution in 2012 [1].

In most cities, vehicular emissions represent the main source of atmospheric pollutants, and both short- and long-term exposure to traffic pollution have been associated with adverse health effects [2,3]. Several studies [4,5,6] have shown an association between lung function decline and long-term exposure to air pollution in adults. Evidence that reduction in air pollution may improve lung function [7], as well as attenuate its decline with age [8], is also available. In an urban scenario, such as São Paulo, an individual’s exposure to air pollution is highly variable and is dependent on the time spent in traffic, residence location and working conditions. Additionally, different categories of workers demand different physical activities, and the healthy worker effect cannot be assumed for all of them.

With the objective of evaluating the chronic effect of exposure to traffic-generated PM_2.5_ on lung function, we used personal monitors to collect data from outdoor workers with potentially different levels of exposure in São Paulo, Brazil. The use of personal monitors allowed a closer assessment of the actual exposure of individuals, compared with fixed stations, because it takes into account the periods of outdoor exposure times and indoor exposure times (inside the homes and workplaces) and, in this study, also inside vehicles (cabs), which have concentrations that may differ from outdoor environments and are usually lower [9,10]. These factors were particularly important to consider for a study conducted in São Paulo, a city with 11 million inhabitants spread over an area of 1,500 Km^2^ with different and irregular traffic and topography.

## Methods

This was an observational study approved by the Ethics on Research Committee (CAPPesq) from the Hospital das Clínicas da Faculdade de Medicina da Universidade de São Paulo (file 0565/07). All participants signed an informed consent form.

### Participants

To explore the effects of exposure variability, three categories of workers were selected. Two of the categories, that is, taxi drivers and traffic controllers, were expected to have greater exposure, whereas the third, that is, forest rangers, were presumably subjected to lower levels of exposure because they spend part of their daily life in a park in the outskirts of São Paulo. The inclusion criteria were as follows: a) working on streets and/or avenues (traffic controllers or taxi drivers) or city parks for at least two years; b) male; c) age between 18 to 65 years; and d) non-smoker or former smoker for only one year.

### Initial Evaluation

Initial evaluation included a recording of demographic and anthropometric characteristics, confirmation of the non-smoker condition, occupational and cardiorespiratory risk assessment, as well as a clinical examination. All participants answered questions on tobacco smoke status and chronic comorbidities. Exhaled CO_2_ (ppm) was determined via a Micro CO Meter (Micro Medical Limited, Rochester, UK).

### Study Protocol

From 2008 to 2012, the participants were scheduled in pairs to attend four visits (once a week for one month) in the Heart Institute of the University of São Paulo Medical School. During each visit, participants submitted to the following: i) an assessment of complications between the visits, ii) an evaluation of exhaled CO_2_ to confirm the non-smoking status, and iii) spirometry. At 7:00 am on the eve of each clinical evaluation, each participant received a lightweight portable battery operated sampler installed in a shoulder bag to be carried all day and kept nearby during sleeping or bathing periods to record real 24-hour environmental exposure.

### Lung Function

Spirometry was performed in the morning with a KoKo spirometer (Pulmonary Data Services Instrumentation Inc., Louisville, USA), according to ATS/ERS recommendations [11,12]. Three maneuvers were carried out, and the best one was chosen and analyzed according to the ATS/ERS criteria [13]. The values of FVC, FEV_1_ and PEF corresponded to the percentage of predicted values computed according to the reference recommended for the Brazilian population [14]. The values of FEV_1_/FVC as well as of the ratio FEF_25-75%_/FVC were also computed from the measured values, and those of the latter were also presented as percentages of the predicted ratio [(FEF_25-75%_/FVC)_measured_/ (FEF_25-75%_/FVC)_predicted_].

### PM_2.5_ concentration evaluation

The sampler, designed by the Harvard School of Public Health, operated at a flow rate of 4 L/min through an impaction plate to obtain a PM_2.5_ cutoff. A silicone catheter connected the air inlet positioned at the volunteer shoulder to the sample inflow tube. A vacuum pump powered by a rechargeable Li-Ion battery (model WR-5000 Aircheck from SKC) and equipped with a flow control and a chronometer provided the necessary continuous air flow during the 24-hour sampling period. A polycarbonate membrane [Whatman filter with a diameter of 37 mm and 0.8 pore size (part number 110809)], installed inside the sampler after the impaction plate retained the sampled particulate matter. This membrane was weighed before and after the sampling process with an accurate 1 μg scale (Mettler Toledo, model UMX2) following a laboratory protocol developed to control temperature and humidity [15], and the total mass of the collected particulate matter was estimated. The daily average concentration of PM_2.5_ was obtained by dividing the total mass by the total sampled air volume.

### Exposure classification

Although high exposure was expected for taxi drivers and traffic controllers and low exposure was expected for forest rangers, an initial data analysis showed an overlap of the observed PM_2.5_ concentrations for these two groups. This is probably due to the individual monitoring of PM_2.5_ that allowed evaluation of pollutant concentrations outside the workplace, providing a more realistic measure of exposure. As the preliminary analysis suggested that the relationship between lung function variables and PM_2.5_ is not linear, the tertiles of all individual measurements of PM_2.5_ were computed, and the participants were classified into three exposure groups: low, when all measures were lower than the first tertile; high, when all measures were larger than the second tertile; and medium, otherwise. This classification ensured that all subjects in the low exposure group were always exposed to low concentrations of PM_2.5_ during the study and that those in the high exposure group were always exposed to higher concentrations of PM_2.5_.

### Statistical Analysis

The statistical analysis was based on the averages of the within-individual spirometric variables. Baseline covariates were accounted for in the analysis.

The means of the baseline and spirometric variables for the three groups were compared with a one-way analysis of variance. When residual diagnostic tools suggested course deviations from the model assumptions, the analysis was complemented by a Kruskal-Wallis test. To assess the association between passive smoking, respiratory symptoms, hypertension and/or diabetes and exposure to air pollution, likelihood ratio tests were considered. The association between spirometric variables and exposure, adjusted for age, waist circumference, time at their job, daily work hours, diabetes or hypertension, environmental tobacco smoke and previous smoking habits, was initially assessed using multiple linear regression fitted by least squares. A residual analysis suggested the existence of outliers and robust regression models based on MM-estimators were considered. The models were fitted via the *lmrob* function from the *robustbase* library in the R software package (R Development Core Team 2013).

## Results

Four subjects were excluded due to inconsistencies in the pulmonary function measurements, resulting in a sample size of 101 (S1 Table).

**Fig 1:**
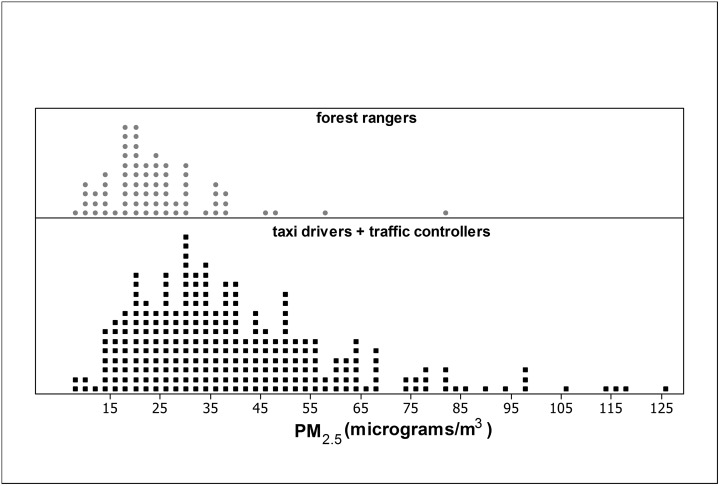
Dot plots of the PM_2.5_ measurements of different occupation groups during the study period.

Overall, 334 measures of PM_2.5_ and 320 pulmonary function tests were completed during the study period. Fig 1 displays the distribution of all 334 individual PM_2.5_ measurements during the study period and shows the overlap of PM_2.5_ concentrations when occupational categories were considered. This overlap and the nonlinearity of the relationship between lung function variables and PM_2.5_ motivated the definition of the exposure groups regarding the PM_2.5_ tertiles, namely, 25 μg/m^3^ and 39.6 μg/m^3^.

### Demographic and clinical characteristics

In Table 1, we present summary statistics for PM_2.5_ and demographic and clinical characteristics according to the exposure groups. On average, participants in the low-exposure group had worked in the same function for a longer period and had lesser work shifts than those in the other groups.

**Table 1:** PM_2.5_ concentration and baseline characteristics of participants (N = 101).

	Exposure groups^(1)^			
Variables	Low (N = 15)	Medium (N = 76)	High (N = 10)	p
PM_2.5_	17.8 (±3.5)	36.6±(10.8)	67.4±(14.1)	<0.001^(2)^
Age (years)	46.6 (±8.4)	48.4 (±9.0)	49.3 (±12.4)	0.730 ^(2)^
BMI (kg/m^2^)	26.4 (±3.7)	28.9 (±4.4)	28.4 (±3.7)	0.117 ^(2)^
Waist circumference (cm)	94.9 (±8.8)	102.5 (±12.7)	101.3 (±10.4)	0.098 ^(2)^
Time of work (years)	16.5 (±7.8)	10.3 (±8.2)	11.9 (±11.4)	0.041 ^(2)^
Work shift (hours)	9.3 (±2.4)	11.3 (±3.5)	12.6 (±3.5)	0.040 ^(2)^
Exhaled Carbon Monoxide (ppm)	2.5 (±2.9)	2.6 (±3.1)	1.5 (±2.0)	0.525 ^(2)^
ETS^(3)^ (N, %)	7 (46.7)	39 (51,3)	4 (40.0)	0.775 ^(4)^
at home (N, %)	1 (6.7)	5 (6.6)	2 (20.0)	0.430 ^(5)^
at work (N, %)	6 (40.0)	41 (54.0)	5 (50.0)	0.610 ^(5)^
Former smoking habits (N, %)	2 (13.3)	21 (28.0)	5 (50.0)	0.135 ^(5)^
Hypertension or diabetes (N, %)	5 (33.3%)	24 (31.6%)	3 (30.0%)	0.984 ^(5)^
Hemoglobin (g/dL)	15.3 (±0.9)	15.1 (±0.9)	14.8 (±0.6)	0.463 ^(2)^
Hematocrit (%)	44.4 (±2.9)	44.7 (±2.4)	43.9 (±1.3)	0.587 ^(2)^
White blood cells (N/mm3)	6217 (±1685)	6765 (±1634)	7052 (±2539)	0.469 ^(2)^
Total Cholesterol (mg/dL)	200.1 (±39.6)	212.1 (±34.2)	201.8 (±34.9)	0.384 ^(2)^
HDL (mg/dL)	47.4 (±8.3)	46.2 (±12.7)	42.9 (±8.3)	0.662 ^(2)^
LDL (mg/dL)	130.3 (±37.6)	136.5 (±31.6)	132.8 (±32.2)	0.774 ^(2)^
Albumin (g/dL)	4.7 (±0.2)	4.7 (±0.2)	4.8 (±0.3)	0.623 ^(2)^
Fasting blood glucose (mg/dL)	96.0 (±9.7)	101.4 (±23.4)	106.3 (±21.7)	0.510 ^(2)^

^(1)^ Low: all measures of PM_2.5_ were lower than 25 μg/m^3^, High: all measures of PM_2.5_ were larger than 39.6 μg/m^3^ and Medium, otherwise;

^(2)^ ANOVA;

^(3)^ Environmental Tobacco Smoke;

^(4)^ Chi-Squared test;

^(5)^ Likelihood- Ratio test; Results expressed as the mean (± standard deviation) or number (percentage).

### Lung Function

**Fig 2:**
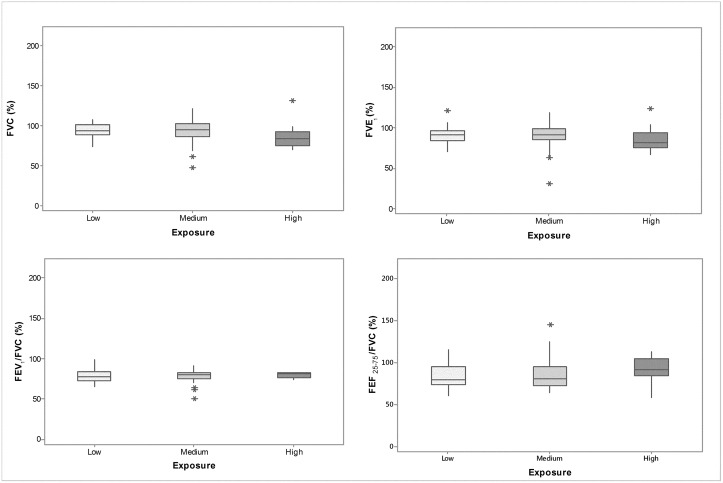
Box-plots of the spirometric parameters of each exposure group. The values of FVC and FEV_1_ correspond to the percentage of predicted values and FEF_25-75%_/FVC, and the ratio of FEV_1_/FVC was computed considering the measured values.

In Table 2, we present descriptive statistics for the lung function data expressed in terms of percentage of the predicted value. In general, we observed a decrease in average FVC and FEV_1_ from the low exposure group to the high exposure group, although without statistical significance. On the other hand, a significant increase in FEF_25-75%_/FVC was observed, without a decrease in the FEV_1_/FVC. Box-plots for the spirometric variables are depicted in Fig 2.

**Table 2:** Mean (standard errors) for the spirometric data of participants and p-values for the comparison of the exposure groups (N = 101).

	Exposure groups ^(1)^			
Spirometric parameters (%)	Low (N = 15)	Medium (N = 76)	High (N = 10)	p
FVC ^(2)^	94.0 (13.0)	94.9 (16.4)	83.5 (17.2)	0.076 ^(3)^
FEV_1_ ^(2)^	91.5 (11.3)	91.2 (13.8)	82.2 (19.2)	0.143 ^(3)^
FEV_1_/FVC	77.5 (10.9)	79.6 (7.3)	80.3 (6.8)	0.997 ^(3)^
FEF_25-75%_/FVC	82.6 (14.0)	84.3 (15.0)	91.0 (16.4)	0.358^(3)^
FEF_25-75%_/FVC ^(2)^	102.5 (17.1)	104.8 (18.3)	120.2 (22.5)	0.041 ^(3)^
PEF ^(2)^	79.9 (30.0)	74.9 (18.5)	72.6 (26.7)	0.386 ^(3)^

^(1)^ Low: all measures of PM_2.5_ were lower than 25 μg/m^3^, High: all measures of PM_2.5_ were larger than 39.6 μg/m^3^ and Medium, otherwise;

^(2)^ Percentage of predicted value;

^(3)^ Kruskal-Wallis test.

In Table 3, we highlight the adverse effects of the level of exposure to PM_2.5_ on the spirometric parameters comparing the high and medium exposure groups to the low exposure group. To verify the robustness of the observed findings, we performed a sensitivity analysis, progressively adding more explanatory variables to check how the estimates were affected across different model specifications. The estimates of expected FVC, FEV_1_, and FEF_25-75%_/FVC remained reasonably stable for the different sets of explanatory variables considered. In the high exposure group, smaller average FVC and FEV_1_ values were observed. Additionally, in the same group, average FEF_25-75%_/FVC values were higher with no statistically significant differences detected for FEV_1_/FVC.

**Table 3:** Effects of PM_2.5_ on lung function in different stages of the regression model fitting: high exposure—low exposure and medium exposure—low exposure.

Spirometric parameters (%)	Exposure groups compared ^(1)^	Model 1			Model 2			Model 3			Model 4		
		Effect	SE^(2)^	p	Effect	SE^(2)^	p	Effect	SE^(2)^	p	Effect	SE^(2)^	p
FVC ^(3)^	Medium -Low	-0.13	2.71	0.960	1.01	2.97	0.730	0.81	3.18	0.800	-0.24	3.01	0.938
	High—Low	-12.96	3.88	0.001	-11.01	3.98	0.007	-10.76	4.09	0.010	-12.19	3.98	0.003
FEV_1_ ^(3)^	Medium -Low	0.94	3.35	0.780	1.77	3.47	0.611	1.76	3.50	0.617	1.16	3.33	0.729
	High—Low	-10.12	5.03	0.047	-9.36	4.52	0.041	-8.41	5.04	0.099	-9.10	5.10	0.078
FEV_1_/FVC	Medium -Low	1.51	2.39	0.529	1.76	3.02	0.561	1.86	3.29	0.572	1.96	3.31	0.557
	High—Low	1.42	2.51	0.573	1.46	3.09	0.638	1.97	3.28	0.550	1.97	3.35	0.558
FEF_25-75%_/FVC	Medium—Low	1.01	3.73	0.787	-0,65	3.23	0.842	-0.78	3.22	0.808	0.049	3.38	0.989
	High—Low	11.02	6.18	0.073	13.01	3.91	0.001	12.29	4.53	0.008	13.34	4.55	0.004
FEF_25-75%_/FVC ^(3)^	Medium -Low	1.74	3.09	0.574	-0.83	3.66	0.821	-0.80	3.79	0.833	-0.91	3.86	0.814
	High—Low	18.43	5.78	0.002	15.57	5.93	0.010	14.99	5.93	0.013	14.86	6.08	0.017
PEF ^(3)^	Medium -Low	-4.45	5.53	0.423	-1.48	5.77	0.799	-1.76	5.82	0.763	-1.84	5.66	0.746
	High—Low	-6.87	7.34	0.351	-3.58	7.43	0.631	-4.34	7.99	0.588	-4.41	7.97	0.581

^(1)^ Low: all measures of PM_2.5_ were lower than μg/m^3^, High: all measures of PM_2.5_ were larger than 39.6 μg/m^3^, and Medium, otherwise;

^(2)^ Standard error;

^(3)^ Percentage of predicted value. Predictors included in the models: Model 1: exposure groups; Model 2: exposure groups + age+ waist circumference + time of work + work shift; Model 3: exposure groups + age+ waist circumference + time of work + work shift + environmental tobacco smoke at home + former smoker; Model 4: exposure groups + age+ waist circumference + time of work + work shift + environmental tobacco smoke at home + former smoker + diabetes or hypertension.

## Discussion

This study suggests a significant impairment in pulmonary function for individuals exposed to ambient levels of traffic pollution. Moreover, the results indicated that, in a complex urban scenario such as the one in São Paulo, there is a marked degree of exposure variability amongst outdoor workers. The observed findings were robust and remained reasonably stable for the different sets of explanatory variables considered.

One of the strengths of this study was the use of personal monitors to evaluate exposure to PM_2.5_, which seems more appropriate to assess daily exposure by including work shift, time while commuting and time at home [9,10]. Assuming none or minimum changes in daily routine of participants, the air pollutant personal monitoring assessment may allow estimates of sub acute and even chronic exposures. Our results reinforced the concept that urban dwellers may experience a considerable variation of exposure, depending on their habits and work characteristics, because the difference between the means of the PM_2.5_ in the high and low exposure groups was almost 50 μg/m^3^. Monitoring personal exposure to PM_2.5_ during different weeks contributed to a perception of the most prevalent exposure profiles of the individuals. PM_2.5_ is technically easy to measure and is the pollutant most consistently associated with damage to human health. Due to its high correlation with other pollutants, it may be regarded as a proxy of a set of pollutants. An additional strength of this study is that pulmonary function was evaluated four times, yielding more reliable measures of the health-related outcomes.

Given all participants lived in São Paulo, a city known to have high levels of air pollution, a group without chronic exposure could not be included in the study. This could be considered a limitation and could suggest an underestimation of the effects of air pollution on lung function.

The three occupational categories showed only slightly different intensities of light physical activity.

The differences in exposures to PM_2.5_ seem to be the relevant cause of adverse health effects.

Most of the studies that have focused on chronic [16,17,18] or acute [17,19] effects of air pollution suggested a reduction in pulmonary function and reported evidence of obstructive pulmonary disorder [4,18], although this topic is still controversial [20]. In a recent European multicenter cohort study (ESCAPE), an association between exposure to NO_2_ and PM_10_ with reduction of FEV_1_ and FVC was observed, mainly in obese individuals. In that study, however, the mean levels of PM_10_ (25 μg/m^3^) were much lower than those observed in São Paulo (40 to 50 μg/m^3^) [21].

A longitudinal study carried out in Germany with women living near major roads detected a significant association between exposure to PM_10_ and reduction of FVC, FVE_1_ and FEV_1_/FVC [18]. A study carried out with 1,391 non-smokers followed up for 16 years [22] detected a decrease of 7.2% in FEV_1_ among those with higher exposure levels, as well as a significant decrease in FEV_1_/FVC.

The results of this study are in agreement with previous studies [6,23] in the sense that air pollution is associated with a reduction in FVC and FEV_1_ without a concomitant reduction in FEV_1_/FVC. The mechanisms responsible for such alterations have not been fully identified. Animal studies [24], as well as autopsy studies with humans [25], conducted in São Paulo indicate that exposure to urban levels of air pollution promotes inflammation and structural remodeling of distal airways and centriacinar destruction of alveolar walls. A similar effect, with reduced FVC and FEV_1_, but no reduction of FEV_1_/FVC has been observed after exposure to ozone, probably due to changes in airway hysteresis compared with parenchymal hysteresis [26].

Recently, a cohort study involving 6,300 participants in the northeastern United States concluded that long-term exposure to PM_2.5_ was associated with lower FEV_1_ and a tendency of lower FVC, but not with a reduction in FEV_1_/FVC [27].

Our study also detected a significant increase in FEF_25-75%_/FVC without a corresponding reduction in FEV_1_/FVC. This can be attributed to a characteristic of fine particles that tend to deposit in the small airways, causing wall inflammation, thickening and remodeling, as suggested by studies using lungs from autopsies [28,29]. It is not possible to exclude the possibility of a restrictive disorder. However, measurements of lung volumes are required to confirm this hypothesis.

Another possible explanation for this pattern of disturbance of respiratory function is the air trapping caused by lower airway diseases, which can promote a reduction in FVC without changes in FEV_1_/FVC, as observed in the study of people exposed to the particles generated by the attacks on the World Trade Center [30]. Therefore, FEF_25-75%_ may be increased in this case because it expresses the measurement of the FVC segment that represents less distal airways where the flow is faster. Due to the lack of measurements on lung volumes, diffusing capacity of the lung for carbon monoxide (D_LCO_) or impulse oscillometry, it is not possible to discriminate mechanisms related to the spirometric results.

A study carried out in India with 300 children living in an industrial area and 300 children living in a green zone estimated a prevalence of restrictive ventilatory disorders of 20.3% and 6.0%, respectively, supporting the hypothesis that exposure to air pollution may be associated with lung function impairment [23]. These findings are also consistent with those reported in a study involving 230 children in Mexico City and 19 in Tlaxcala; the authors detected 60% of interstitial markings in the children of Mexico City in contrast to 0% in Tlaxcala, where the PM and ozone levels are lower [31]. A recently published study comparing pulmonary function of healthy children across the Indian rural-urban continuum and Indian children living in the UK suggested that those living in rural areas in India have lower average values of FVC and FEV_1_, without a reduction in average FEV_1_/FVC, suggesting an association between nutritional and environmental effects and restrictive disorders because exposure to biomass in rural areas in India is common [32].

In one of the few studies reporting values of FEF_25-75%_, a restrictive pattern of lung function disorder very similar to that observed in our study was observed in traffic controllers in India [33]. However, in addition to the methodological problems of that study, this effect remains controversial given that a study conducted in the same country three years previously showed reductions in FVC and FEV_1_, but not differences in FVC/FEV_1_ and FEF_25-75%_; however, a reduction in forced inspiratory flow at 50% of inhaled volume (FIF50%) was observed among non-smoking participants [34]. In this study [34], when only smokers were taken into account, smaller values of FEF_25-75%_ and maximum voluntary ventilation among the traffic exposed participants were observed, suggesting a mixed ventilatory disorder [34]. It is already known that some of the people exposed to tobacco smoke develop interstitial lung disease [35,36], which also limits the interpretation of such results.

Experimental studies in animals and humans provide biological plausibility for the association between air pollution and interstitial lung disease, involving oxidative stress, telomere shortening and inflammation mediated by transforming growth factor-β (TGF-β), as shown in a recent review [37].

A limitation of this study is that the number of pack years of former smokers was not assessed. Furthermore, the number of former smokers in the low and high exposure groups was very small, jeopardizing an evaluation of a possible interaction effect between previous exposure to tobacco and vulnerability due to exposure to air pollution.

In conclusion, this study indicated that adult inhabitants of a large urban center, usually exposed to higher concentrations of PM_2.5_ generated from vehicular sources, showed significant changes in lung function (reduction of FVC without changes in FEV_1_/FVC), which do not suggest an obstructive disorder. However, complementary studies are necessary to clarify the mechanisms involved in these observed functional pulmonary alterations.

### What is already known on this subject?

- Pulmonary function is an indicator of mortality risk.
- Exposure to air pollutants is associated with a decline in pulmonary function.
- Studies have shown divergent results to explain this effect and the characteristic of the impairment (restrictive or obstructive).

### What this study adds?

- The environmental exposure to fine particles, generated mainly by the automotive fleet promotes a ventilatory impairment with a reduction of FVC and FEV_1_, but without changes of FEV_1_/FVC and with an increase of FEF_25-75%_.
- These results reinforce the necessity of a more restrictive air quality standard and the implementation of a widely distributed clean public transportation system in large urban centers such as São Paulo.

## Supporting Information

S1 Table Santos et al_PONE-D-16-01680_REV2016_05_23_Data Set. (XLSX)
